# A Risk Warning Model for Anemia Based on Facial Visible Light Reflectance Spectroscopy: Cross-Sectional Study

**DOI:** 10.2196/64204

**Published:** 2025-02-14

**Authors:** Yahan Zhang, Yi Chun, Hongyuan Fu, Wen Jiao, Jizhang Bao, Tao Jiang, Longtao Cui, Xiaojuan Hu, Ji Cui, Xipeng Qiu, Liping Tu, Jiatuo Xu

**Affiliations:** 1 Traditional Chinese Medicine College, Shanghai University of Traditional Chinese Medicine, No. 1200 Cailun Road, Pudong New Area, Shanghai, 201203, China, 86 021 51322143; 2 Clinical Research Unit, Shanghai Municipal Hospital of Traditional Chinese Medicine, Shanghai, China; 3 Department of Hematology, Shanghai Municipal Hospital of Traditional Chinese Medicine, Shanghai, China; 4 Collaborative Innovation Center for Traditional Chinese Medicine Health Services, Shanghai University of Traditional Chinese Medicine, Shanghai, China; 5 School of Computer Science and Technology, Fudan University, Shanghai, China

**Keywords:** anemia, hemoglobin, spectroscopy, machine learning, risk warning model, Shapley additive explanation

## Abstract

**Background:**

Anemia is a global public health issue causing symptoms such as fatigue, weakness, and cognitive decline. Furthermore, anemia is associated with various diseases and increases the risk of postoperative complications and mortality. Frequent invasive blood tests for diagnosis also pose additional discomfort and risks to patients.

**Objective:**

This study aims to assess the facial spectral characteristics of patients with anemia and to develop a predictive model for anemia risk using machine learning approaches.

**Methods:**

Between August 2022 and September 2023, we collected facial image data from 78 anemic patients who met the inclusion criteria from the Hematology Department of Shanghai Hospital of Traditional Chinese Medicine. Between March 2023 and September 2023, we collected data from 78 healthy adult participants from Shanghai Jiading Community Health Center and Shanghai Gaohang Community Health Center. A comprehensive statistical analysis was performed to evaluate differences in spectral characteristics between the anemic patients and healthy controls. Then, we used 10 different machine learning algorithms to create a predictive model for anemia. The least absolute shrinkage and selection operator was used to analyze the predictors. We integrated multiple machine learning classification models to identify the optimal model and developed Shapley additive explanations (SHAP) for personalized risk assessment.

**Results:**

The study identified significant differences in facial spectral features between anemic patients and healthy controls. The support vector machine classifier outperformed other classification models, achieving an accuracy of 0.875 (95% CI 0.825-0.925) for distinguishing between the anemia and healthy control groups. In the SHAP interpretation of the model, forehead-570 nm, right cheek-520 nm, right zygomatic-570 nm, jaw-570 nm, and left cheek-610 nm were the features with the highest contributions.

**Conclusions:**

Facial spectral data demonstrated clinical significance in anemia diagnosis, and the early warning model for anemia risk constructed based on spectral information demonstrated a high accuracy rate.

## Introduction

### Background

Anemia is a condition characterized by a decrease in red blood cell count or hemoglobin concentration below a certain threshold, thereby reducing the blood’s oxygen-carrying capacity throughout the body. It serves as an indicator of poor nutritional and health status [1]. A study conducted from 1990 to 2019 reported that more than 1.7 billion people worldwide were anemic [2]. Typical symptoms include fatigue, drowsiness, weakness, tachycardia, shortness of breath, increased heart rate, decreased appetite, hypotension, and dizziness [3]. According to the results of China’s fourth nutritional survey, the prevalence of anemia among Chinese residents is 20.1%, and the global prevalence of anemia in all ages is 22.8% in 2019 [4], indicating it has become an important public health issue. In a survey of 13,175 Chinese adults aged >50 years, the prevalence of anemia is 31%, indicating that anemia is particularly prevalent in China’s middle-aged and older adult population [5]. Especially among older adults, anemia has been identified as one of the independent risk factors for poor patient outcomes [6]. Studies have found that older adults with anemia were more than twice as likely to suffer from frailty [7], and the survival rate of older adult patients with anemia was significantly lower than that of the nonanemic older adult population [8]. Hemoglobin (Hb) concentration is a reliable indicator for diagnosing anemia [9]. Specifically, a low hemoglobin concentration is strongly correlated with disability, poor physical performance, and declining cognitive ability and strength in aging individuals [10,11], especially so for the oldest individuals [12]. Anemia is associated with a variety of diseases for which there is a negative impact on the patient’s prognosis [13]. Additionally, preoperative anemia has been identified as an independent correlation factor of increased postoperative complications and mortality rates [14]. Anemia is often diagnosed by an invasive blood specimen collection method that patients may find uncomfortable, especially for those who are sensitive and fearful of invasive blood tests. Bateman et al [15] conducted a prospective, multicenter, epidemiological, and observational study with 977 children enrolled and found that blood draws accounted for 73% of daily blood loss, and that 96.5% of the children lost blood as a result of blood draws. In fact, frequent drawing of blood can lead to medical anemia.

### Current Research on Noninvasive Hemoglobin Detection

Current noninvasive testing methods being explored include photoelectric volumetric pulse wave, spectroscopy, and hyperspectral techniques. For diagnosis via photoelectric volumetric pulse wave, Acharya et al [16] developed a multimodel stacking regressor and estimate the total Hb using noninvasively acquired photoplethysmogram. Spectroscopic techniques explore material properties through the interaction between matter and electromagnetic waves of different frequencies [17]. Hyperspectral imaging techniques provide a more detailed segmentation in spectral dimensions and contain much more information than red, green, and blue [18]. Color measurement in Chinese medicine color diagnosis by visible reflectance spectroscopy is similar to clinical color diagnosis, reflecting the accuracy, authenticity, and reliability of color diagnosis results [19]. Raman spectroscopy can be used to analyze the distribution of metabolite, lipid, protein, water, and blood content in tissues by detecting the skin[20]. Hyperspectral imaging can estimate Hb concentration and blood oxygen saturation by diffuse reflection of tissues [21]. Spectral imaging of sublingual microcirculation was proven to be able to detect anemia [22]. Research on noninvasive Hb measurement using spectroscopy predominantly focuses on areas such as the palm, fingernails, and conjunctiva. Kesarwani et al [23] proposed a noninvasive palm pallor–based anemia detection system, which utilizes a smartphone app to estimate Hb levels by monitoring changes in palm pallor. The system achieved a sensitivity of 0.93, mean squared error of 0.701, and root mean squared error of 0.698. Liu et al [24] used a fingertip measurement method, constructed partial least squares and back propagation artificial neural network models, and developed a portable prototype for their noninvasive Hb detection system. Mannino et al [25] used the color of patients’ nail beds from smartphone photos to estimate Hb levels, defining anemia as 11.0 g dL<threshold. The model for the study had a sensitivity and specificity of 0.92 and 0.76 in the classification of anemia versus healthy individuals. Bevilacqua et al [26] proposed an alternative method for noninvasive Hb estimation based on image analysis of specific regions of the conjunctiva; in total, 77 patients with anemia and healthy individuals were studied and modeled using a binary support vector machine (SVM) classifier with a resulting accuracy of 0.844, specificity of 0.824, and sensitivity of 1.000. Kalantri et al [27] conducted a blind, independent comparison of the presence and absence of pallor in physical features (including conjunctiva, tongue, palms, and nail beds) and a reference standard (Hb estimated by an electronic cell counter). Diagnostic accuracy was measured by calculating likelihood ratios, 95% confidence intervals for different Hb thresholds, and the area under the characteristic curve of the subjects. The area under the characteristic curve was 0.84 and 0.71 for Hb of 7 g/dL and 9 g/dL as cutoffs. Wang et al [28] developed HemaApp, which uses a smartphone camera and different light sources to noninvasively monitor Hb concentration based on photoelectric volumetric pulse wave techniques. HemaApp was evaluated on 31 patients, with a light source passing through the patient’s finger for chromatic analysis to estimate the Hb concentration by blood color. Regression analysis of Hb concentration was performed in the paper for different light sources: when white +970 nm LEDs were used*, R*=0.69; when white +970 nm LEDs + incandescent were used, *R*=0.74; when white + 880 nm and 970 nm LEDs + incandescent were used, *R*=0.82.

Facial diagnosis is an important and intuitive method of traditional Chinese medicine (TCM). The facial features of patients with anemia always differ from those of healthy persons, usually showing a pale complexion [27]. Pallor of the conjunctiva, palms, nail beds, or any part of the body is associated with significantly lower Hb concentrations [29]. The continuous development of intelligent diagnostic methods has provided classification models for recognizing facial complexions, which to some extent avoids the subjective limitations of the traditional facial diagnosis that are qualitative and experience-based [30]. An increasing number of experts are integrating color optical theories and modern instrumentation into the modern research field of TCM diagnosis, making the research more scientific and objective, and avoiding biases caused by human factors [31]. However, there is currently a lack of formal studies focusing on noninvasive, spectral Hb monitoring of the facial region [32].

### Goals

Based on this, we collected data from patients with anemia and healthy controls, analyzed the spectral reflectance and image features of their faces, and built an anemia classification model based on machine learning (ML) methods. The results showed that patients with anemia generally have a paler complexion, with the most noticeable differences located in the nose, right cheek, right zygomatic, and jaw.

## Methods

### General Description of Participants

To control for variables such as age, locale, and gender, we implemented rigorous matching criteria. Between March 2023 and September 2023, we collected data on people who underwent routine physical examinations at the Shanghai Jiading and Gaohang Community Health Centers, from which we randomly selected 78 healthy individuals, consisting of 38 males (49%) and 40 females (51%), aged 45 to 85 years, with a mean age of 68 years. Additionally, from August 2022 to September 2023, we gathered data from patients admitted to the hematology ward at the Shanghai Hospital of Traditional Chinese Medicine, ultimately analyzing 78 patients with anemia consisting of 38 males (49%) and 40 females (51%) aged 45 to 85 years, with a mean age of 67 years. This anemia group contained 26 mild (33%), 26 moderate (33%), and 26 severe cases (33%) of anemia.

### Diagnostic Criteria

According to the diagnostic criteria established by Chinese hematologists [33], in China, anemia can be diagnosed in adult males with a Hb level ≤120 g/L and in adult females (nonpregnant) with Hb ≤110 g/L. Within this range, Hb ≥90 g/L is considered mild anemia, 60 g/L≤ Hb ≤89 g/L is considered moderate anemia, and 30 g/L≤Hb ≤59 g/L is considered severe anemia.

### Inclusion and Exclusion Criteria

The inclusion criteria for the healthy control group is as follows: (1) the absence of any acute or chronic diseases according to clinical diagnostic criteria (no diagnosis of any acute disease within 3 months, and no diagnosis of any chronic disease within 6 months), (2) no current medication use or other irregularity found in the blood, and (3) regular urine, liver, and kidney functions.

The inclusion criteria for the anemia group is as follows: (1) the patient’s Hb value must meet the diagnostic criteria for anemia [33]; (2) the patient exhibits symptoms of anemia, such as pale complexion, lips, or nails, fatigue, weakness, shortness of breath, and resting tachycardia [3,34]; and (3) the patient’s age is between 45 and 85 years. Both male and female patients were included.

The exclusion criteria for the anemia group is as follows: (1) the patient requests to withdraw informed consent; (2) the patient is unable to meet the inclusion criteria; (3) presence of severe cardiovascular, cerebrovascular, endocrine, motor, autoimmune, or infectious diseases; (4) presence of mental illnesses or disorders of consciousness and communication; (5) patients undergoing blood transfusion; and (6) pregnant and breastfeeding women, or those preparing for pregnancy.

### Observation Methods

**Figure 1.**
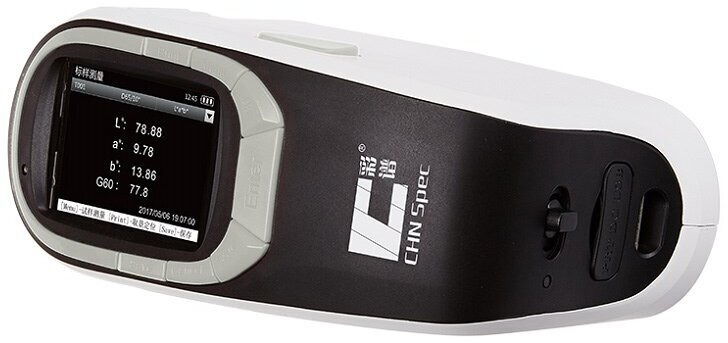
CS-600CG Spectrophotometer

Facial spectroscopic data of the healthy controls and patients with anemia were collected using a CS-600CG spectrophotometric colorimeter (Figure 1).

To obtain more comprehensive facial information, we selected 8 points on the subjects’ faces: the forehead, glabellum, nose, jaw, right zygomatic, left zygomatic, right cheek, and left cheek. It should be noted that subjects were required to be free of cosmetics and maintain a natural facial complexion during data collection. The following specific steps were taken: (1) calibrate the spectrometer and adjust the parameters to standard values; (2) instruct subjects to keep their faces clean; (3) sterilize the instrument with 75% medical alcohol; (4) position the collection port against the subject’s skin and collect data at the following locations in the order specified: forehead, glabellum, nose, jaw, right zygomatic, left zygomatic, right cheek, and left cheek; and (5) verify the consistency of the collected data and minimize excessive variability between each facial area.

### Statistical Analysis

We used SPSS (version 25.0; IBM Corp) for statistical analysis. Data satisfying normality were expressed as mean (SD), whereas data not satisfying normality were expressed as median (IQR). The significance level was set at *α*=.05. To compare the 2 groups, independent samples *t* tests were used for data that were normally distributed and demonstrated homogeneity of variance; otherwise, Mann-Whitney *U* tests were applied. All tests were 2-tailed, and a *P*<.05 was considered to indicate statistical significance. To compare the 4 groups, 1-way ANOVAs were used for data that were normally distributed and exhibited homogeneity of variance; Kruskal-Wallis tests were applied for data that were not normally distributed or exhibited inhomogeneity of variance. All tests were 2-tailed, and a *P*<.05 was considered to indicate statistical significance.

### Model Selection

After selecting feature factors from all independent variables, we divided patients with anemia and healthy controls into training and test sets. Multiple ML classification models were then applied for a comprehensive analysis to compare the discriminative performance of different models. Additionally, we evaluated and validated the results using the optimized model. A SHAP demonstration model was also developed. The specific steps are as follows:

1. Feature selection: Initially, we conducted the least absolute shrinkage and selection operator (LASSO) regression analysis in R to adjust variable selection and complexity. Subsequently, the results from the LASSO regression analysis were utilized as feature variables for machine learning.

2. Data division: To prevent randomness in modeling results, we classified patients with mild, moderate, and severe anemia according to the diagnostic criteria established by Chinese hematologists. Using average random sampling, we divided the data proportionally for training and validating the classification model: 108 cases (70%) for the training set and 48 cases (30%) for the test set.

3. Comprehensive analysis of multiple classification models: We utilized an open-source version of Python 3.7 for our analysis. Subsequently, we used various functions from the scikit-learn library via Python 3.7, applying LASSO regression to extract different spectral bands as feature bands. We constructed a risk warning model for anemia using 10 ML algorithms: logistic regression, decision tree, SVM, random forest, k-nearest neighbor (KNN), artificial neural network (ANN), Bayesian classifier, extreme gradient boosting (XGBoost), adaptive boosting (AdaBoost), and light gradient boosting machine (LightGBM).

Logistic regression is used for binary and multiclass classification, using maximum likelihood for parameter estimation [35]. Decision tree uses tree diagrams for decision-making, handling classification and regression without data standardization [36]. SVMs find optimal hyperplanes for classification, using kernel tricks for complex issues [37]. Random forest uses multiple trees for better stability and accuracy, ideal for high-dimensional datasets [38]. KNN predicts by nearest categories, which is best for simple, small-scale datasets [39]. ANNs learn complex data relationships through neural connections, great for nonlinear and large datasets [40]. Bayesian classifiers use Bayes’ theorem for probabilistic outputs, suitable for medical diagnostics [41]. XGBoost optimizes boosting trees for performance and efficiency, excelling in large or feature-rich datasets [42]. AdaBoost improves classification by adjusting for misclassified samples, using multiple weak classifiers [43]. LightGBM focuses on efficient learning with high precision and speed, and it is optimized for distributed environments [44]. We then trained and tested the above parameterized models, analyzed the importance of the training set and testing set indicators in different models, and selected the optimal one. Python 3.7 was used to construct the area under the receiver operating characteristic curve (AUROC), which is often used to describe tools for diagnostic testing or the identification accuracy of predictive models [45]. Python 3.7 was used to plot precision-recall (PR) curves, which were widely used to evaluate the performance of models. PR and the area under the PR (average precision [AP]) curve can provide a valuable complement to existing model evaluation methods [46]. Python 3.7 was used to plot the decision curve analysis (DCA), essentially the decision analysis. Thus, with these evaluations, it was possible to determine the viability of a given model, as well as which of several models was optimal, with significant advantages in assessing the clinical applicability of the model [47]. Also, we evaluated the performance of the classification models using a range of metrics, including accuracy, sensitivity, specificity, *F*_1_-score, AUROC, positive predictive value (PPV), and negative predictive value (NPV). The formulas for accuracy, sensitivity, specificity, precision, *F*_1_-score, PPV, and NPV are as follows:


(1) $$Accuracy=\frac{TP+TN}{TP+TN+FP+FN}$$

(2) $$Sensitivity=\frac{TP}{TP+FN}$$

(3) $$Specificity=\frac{TN}{TP+FP}$$

(4) $$Precision=\frac{TP}{TP+FP}$$

(5) $$F_{1}=\frac{2\times Precision\times Sensitivity}{Precision+Sensitivity}$$

(6) $$PPV=\frac{TP}{TP+FP}$$

(7) $$NPV=\frac{TN}{TN+FN}$$

4. We used SHAP library via Python 3.7 to plot the SHAP interpretation of importance and contribution to the model and interpreted the model results by calculating the contribution of each feature to the prediction results.

### Ethical Considerations

The studies involving human participants were reviewed and approved by the Ethics Committee of Shanghai Municipal Hospital of Traditional Chinese Medicine affiliated to Shanghai University of Traditional Chinese Medicine (registration number 2021SHL-KY-03-01) and the institutional review board of Shuguang Hospital affiliated with Shanghai University of Traditional Chinese Medicine (registration number 2018-626-55-03). Informed consent was obtained from all subjects involved in the study. The privacy and confidentiality of patients' personal information were strictly protected throughout the research process. All patient data were anonymized and stored securely.

## Results

### Participant Characteristics

The baseline characteristics of the first 2 healthy and patient groups are shown in Table 1 and the baseline characteristics of the latter healthy and patient groups classified by severity group are shown in Table 2.

**Table 1.** Baseline comparison of 2 groups (n=156).

Characteristics	Healthy controls (n=78)	Patients with anemia (n=78)	*P* value
Gender, n (%)			
Male	38 (49)	38 (49)	1.00
Female	40 (51)	40 (51)	
Age (years), mean (SD)	67.03 (10.62)	68.24 (4.68)	.94

**Table 2.** Baseline comparison of 4 groups (n=104).

Characteristics	Healthy controls (n=26)	Anemia patients (n=78)			*P* value
		Mild (n=26)	Moderate (n=26)	Severe (n=26)	
Gender, n (%)					
Male	13 (50)	12 (46)	14 (54)	12 (46)	.94
Female	13 (50)	14 (54)	12 (46)	14 (54)	
Age (years), mean (SD)	67.03 (10.62)	70.35 (11.03)	65.19 (11.14)	65.54 (9.19)	.11

The results indicate that there were no statistically significant differences in gender and age distribution among the 2 or 4 groups (*χ*^*2*^ test for gender, Kruskal-Wallis test for age).

### Statistical Analysis of Facial Spectral Data

The results (Multimedia Appendix 1) showed statistically significant differences in spectral reflectance at specific wavelength ranges between the 2 groups (*P*<.05), including the forehead (400‐600 nm), glabellum (400‐700 nm), nose (400‐700 nm), jaw (400‐610 nm), right zygomatic (400‐700 nm), left zygomatic (400‐470 nm and 500‐610 nm), and right cheek (400‐700 nm). Additionally, significant differences were noted at the forehead (400‐420 nm, 520‐590 nm, 690‐700 nm), glabellum (400‐700 nm), nose (400‐700 nm), jaw (470‐500 nm), right zygomatic (400‐700 nm), left zygomatic (440‐700 nm), right cheek (400‐700 nm), and left cheek (400‐600 nm, 650‐700 nm) between the 4 groups (*P*<.05).

**Figure 2.**
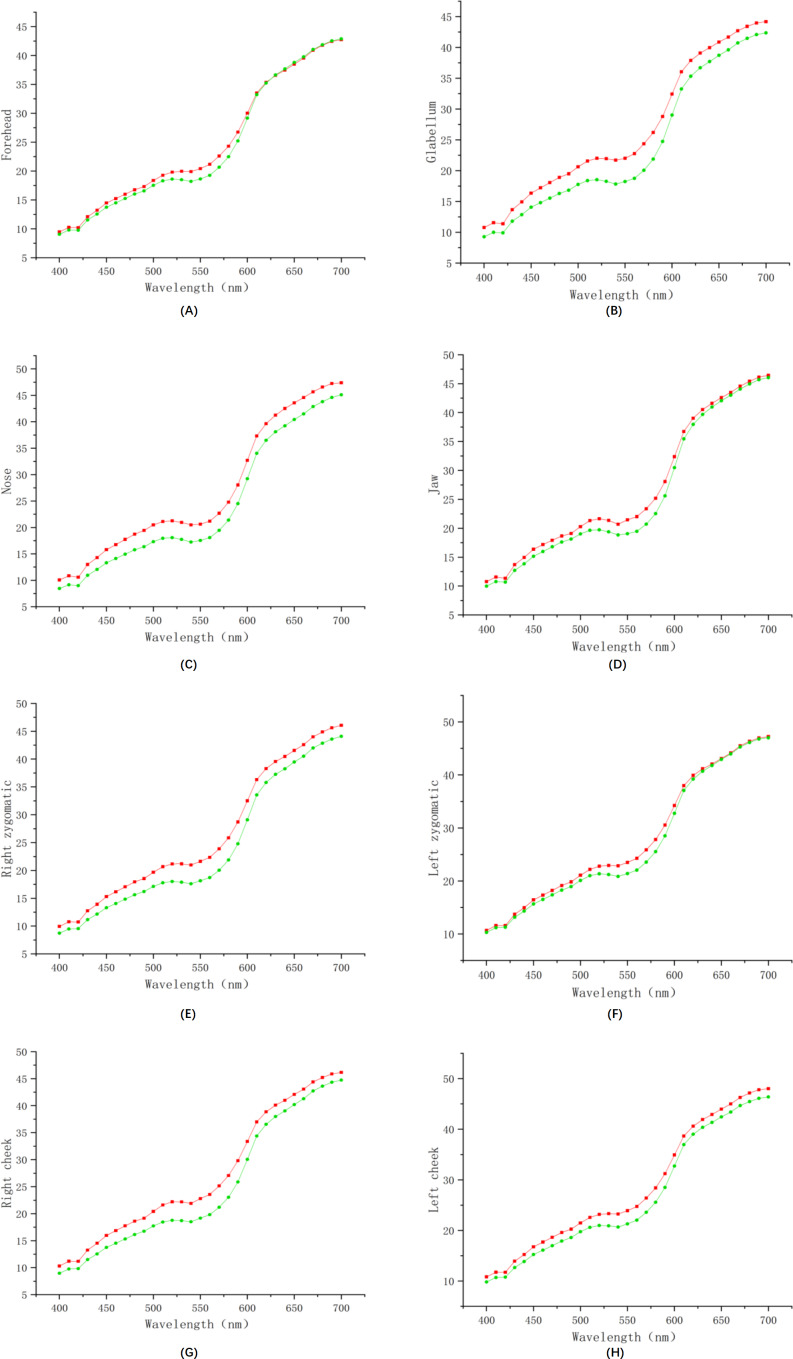
Comparison between 2 groups of spectral lines for 8 positions: (**A**) the spectral reflectance of the forehead, (**B**) the spectral reflectance of the glabellum, (**C**) the spectral reflectance of the nose, (**D**) the spectral reflectance of the jaw, (**E**) the spectral reflectance of the right zygomatic, (**F**) the spectral reflectance of the left zygomatic, (**G**) the spectral reflectance of the right cheek, and (**H**) the spectral reflectance of the left cheek.

To directly observe and understand the differences in facial spectral images between the anemia group and the healthy group, we plotted the average spectra at 8 locations for both the anemic and healthy groups, as depicted in Figure 2. The red line represents the average spectral reflectance of the anemic group, while the green line represents that of the healthy control group. It is evident that at the glabellum, nose, right zygomatic, and right cheek locations, the spectral reflectance of the anemic group is significantly higher than that of the healthy control group. Similarly, at the forehead, jaw, left zygomatic, and left cheek locations, the spectral reflectance of the anemic group remained significantly higher, particularly in the 480‐630 nm range.

**Figure 3.**
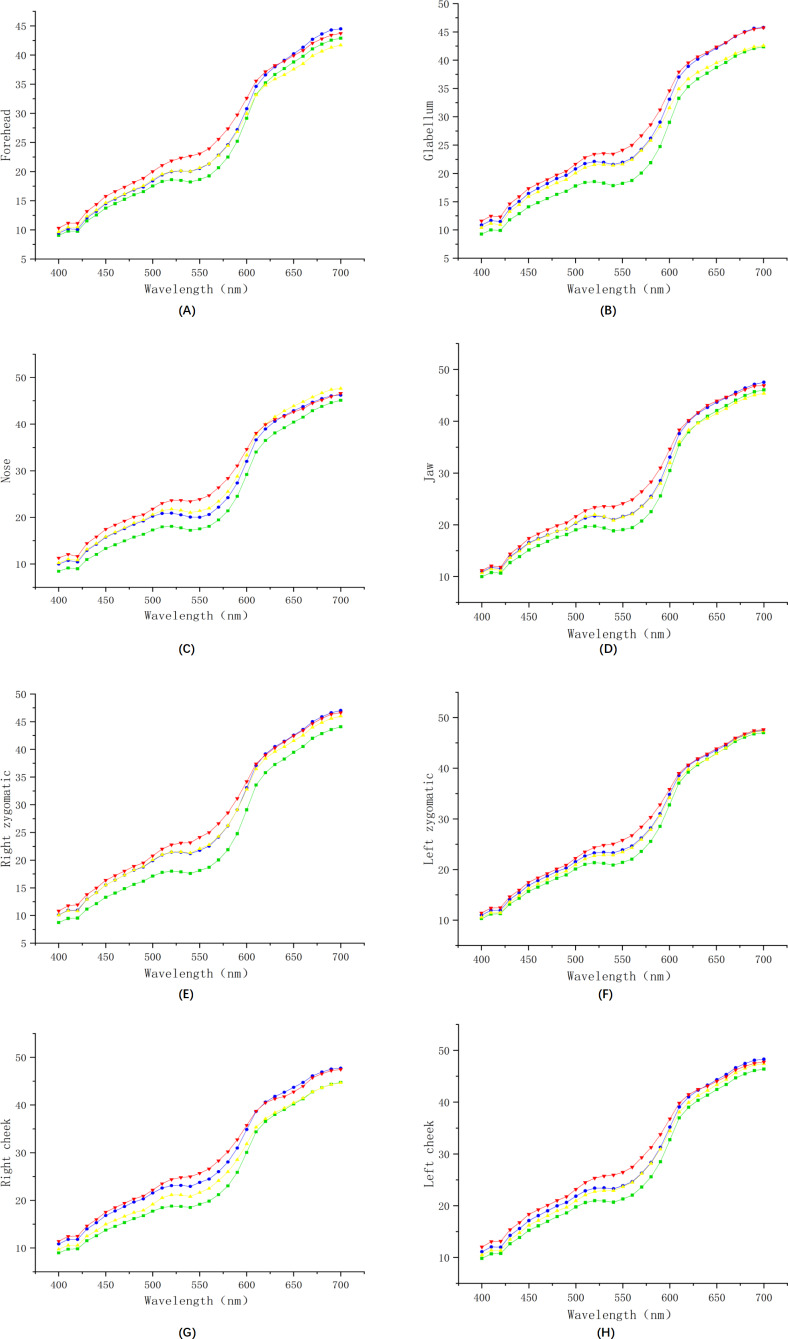
Comparison between 4 groups of spectral lines for 8 positions: (**A**) the spectral reflectance of the forehead, (**B**) the spectral reflectance of the glabellum, (**C**) the spectral reflectance of the nose, (**D**) the spectral reflectance of the jaw, (**E**) the spectral reflectance of the right zygomatic, (**F**) the spectral reflectance of the left zygomatic, (**G**) the spectral reflectance of the right cheek, and (**H**) the spectral reflectance of the left cheek.

Furthermore, to directly observe and understand the differences in facial spectral images between the anemia group and the healthy control group, we plotted the average spectra of the healthy control group and the mild, moderate, and severe anemia groups across a total of 4 groups at 8 positions, as shown in Figure 3. The red line represents the average spectral reflectance of the moderate anemia group, the yellow line represents that of the severe anemia group, the blue line represents the spectral reflectance of the mild anemia group, and the green line represents the average spectral reflectance of the healthy control group.

We observed that the spectral reflectance of both the healthy control group and the severe anemia group is significantly different from that of the other groups at all 8 positions. Moreover, a gradual decrease in spectral reflectance is noted from the severe anemia group to the moderate anemia group, then to the mild anemia group, and finally to healthy individuals across the 400‐630 nm wavelength interval. This gradient of decrease was particularly pronounced at the nose.

### Screening of Characteristic Factors for Risk of Patients With Anemia

**Figure 4.**
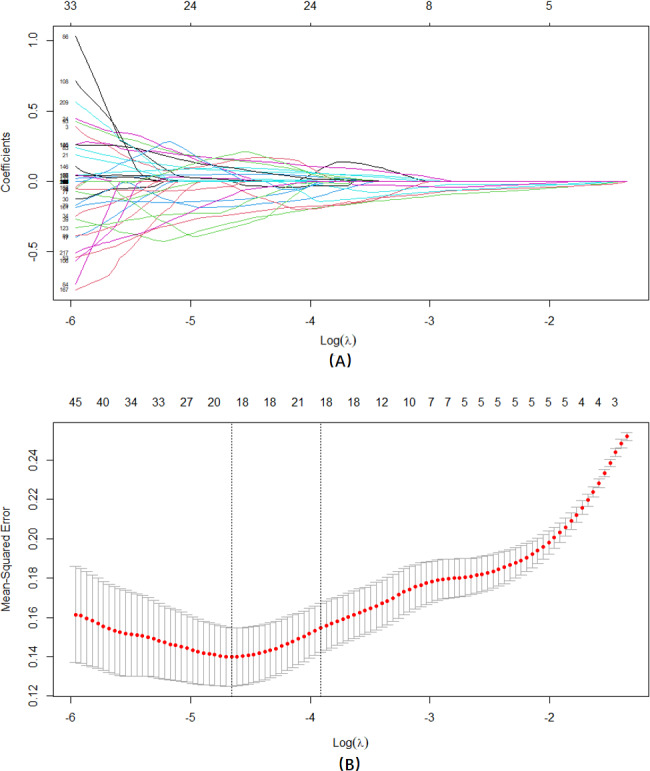
LASSO regression analysis was used to select characteristic factors. (**A**) The use of 10-fold cross-validation to draw vertical lines at selected values, where the optimal lambda produces 20 nonzero coefficients. (**B**) In the LASSO model, the coefficient profiles of 217 features were drawn from the log (λ) sequence. Vertical dotted lines are drawn at the minimum mean square error (λ=0.009) and the standard error of the minimum distance (λ=0.020). LASSO: least absolute shrinkage and selection operator.

Using the presence or absence of anemia as the dependent variable, we performed LASSO regression analysis on 217 independent variables exhibiting statistical differences (Figure 4). LASSO can compress variable coefficients to prevent overfitting and address severe collinearity issues [48]. The results showed that (lambda with minimum mean square error=0.009) 217 independent variables were reduced to 20 (20/217, 9%). The selected variables included glabellum-570 nm, right cheek-520 nm, right zygomatic-570 nm, jaw-570 nm, left cheek-610 nm, left cheek-700 nm, nose-700 nm, jaw-490 nm, nose-490 nm, left zygomatic-500 nm, jaw-610 nm, left cheek-420 nm, forehead-420 nm, nose-400 nm, right cheek-640 nm, right zygomatic-670 nm, glabellum-660 nm, glabellum-670 nm, jaw-420 nm, and glabellum-420 nm.

### Comprehensive Analysis of Classified Multimodel

We included locations and bands that exhibited statistically significant differences as identified in our statistical analyses and used these to construct various models. Specifically, we used logistic regression, decision tree, SVM, random forest, KNN, ANN, Bayesian classifier, XGBoost, AdaBoost, and LightGBM algorithms.

By using both the anemia group and the healthy control group as evaluation test sets, we evaluated the performance of the 10 ML methods, with the results summarized in Table 3.

**Table 3.** Classification model results of each model (95% CIs).

Classifier	Precision	Specificity	Sensitivity	*F*_1_-score	PPV^a^	NPV^b^	Accuracy	AUC^c^	AP^d^
LR^e^	0.836-0.924	0.831-0.919	0.871-0.962	0.853-0.943	0.836-0.924	0.867-0.959	0.846-0.946	0.889-0.983	0.874-0.966
DT^f^	0.887-0.980	0.910-1.000	0.554-0.613	0.682-0.754	0.887-0.980	0.662-0.732	0.721-0.821	0.835-0.922	0.758-0.838
SVM^g^	0.864-0.955	0.871-0.963	0.792-0.875	0.826-0.913	0.864-0.955	0.804-0.888	0.825-0.925	0.925-1.000	0.916-1.000
RF^h^	0.867-0.959	0.870-0.963	0.831-0.919	0.849-0.938	0.867-0.959	0.836-0.924	0.846-0.946	0.879-0.972	0.798-0.882
KNN^i^	0.897-0.992	0.910-1.000	0.673-0.744	0.783-0.833	0.897-0.992	0.728-0.805	0.783-0.883	0.871-0.963	0.825-0.912
ANN^j^	0.809-0.894	0.792-0.875	0.910-1.000	0.846-0.946	0.809-0.894	0.905-1.000	0.846-0.946	0.905-1.000	0.890-0.983
NB^k^	0.769.-0.850	0.792-0.875	0.673-0.744	0.718-0.793	0769.-0.850	0.704-0.778	0.721-0.821	0.811-0.897	0.775-0.856
XGBoost^l^	0.855-0.945	0.871-0.963	0.712-0.788	0.777-0.859	0.855-0.945	0.746-0.825	0.783-0.883	0.849-0.939	0.774-0.855
AdaBoost^m^	0.774-0.856	0.752-0.831	0.871-0.963	0.820-0.906	0.774-0.856	0.860-0.950	0.804-0.904	0.902-0.997	0.869-0.960
LightGBM^n^	0.712-0.788	0.712-0.788	0.712-0.788	0.712-0.788	0.712-0.788	0.712-0.788	0.700-0.800	0.833-0.921	0.806-0.891

a PPV: positive predictive value.

b NPV: negative predictive value.

c AUC: area under the curve.

d AP: average precision.

e LR: logistic regression.

f DT: decision tree.

g SVM: support vector machine.

h RF: random forest.

i KNN: k-nearest neighbor.

j ANN: artificial neural network.

k NB: naive Bayes.

l XGBoost: extreme gradient boosting.

m AdaBoost: adaptive boosting.

n LightGBM: light gradient boosting machine.

**Figure 5.**
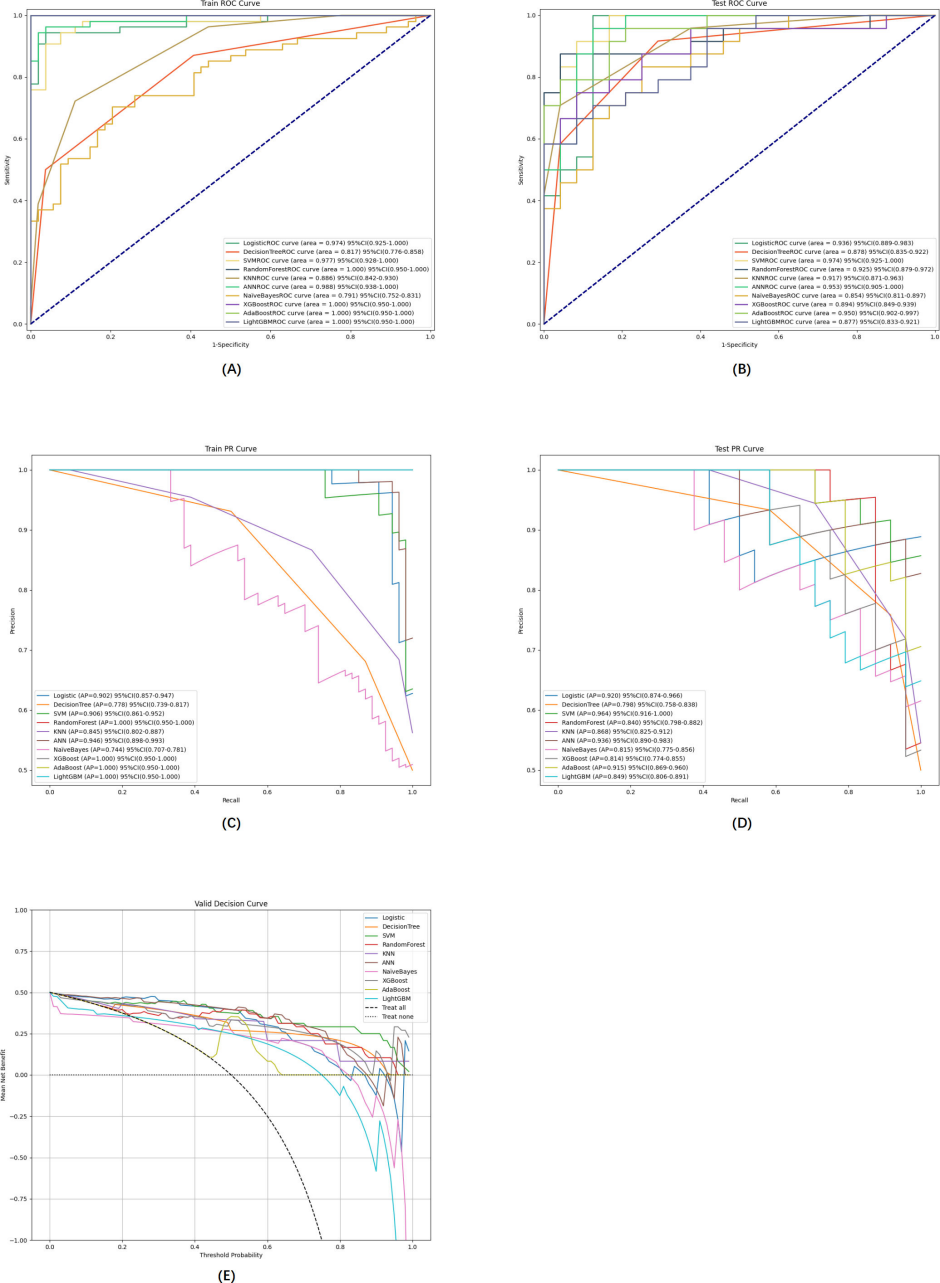
Comprehensive analysis of 10 machine learning models. (**A**) Training set ROC curve and (AUC), (**B**) test set ROC and AUC, (**C**) training set PR curves and AP, (**D**) test set PR curves and AP. The y-axis is the precision axis and the x-axis is the recall axis. If the PR curve of one model is completely covered by the PR curve of another model, the latter can be considered superior to the former; the higher the AP value, the better the model performance. (**E**) Test set DCA, where the black dashed line represents the hypothesis that all subjects are anemic, and the red dashed and thin black lines represent the hypothesis that all subjects are healthy. The remaining solid lines represent different models. The different colors in the pictures represent the corresponding models and the values are expressed as mean and 95% CI. AP: average precision; AUC: area under the curve; DCA: decision curve analysis; PR: precision-recall; ROC: receiver operating characteristic.

To ensure that we considered both the precision and clinical applicability of our predictive models, we plotted the receiver operating characteristic (ROC) curve (Figure 5A and B), the PR curve (Figure 5C and D), and the DCA curve (Figure 5E). A comprehensive comparison indicated that the SVM prediction model demonstrated superior performance, with an accuracy of 0.875 (95% CI 0.825-0.925), an AUC of 0.974 (95% CI 0.925-1.000), and an AP of 0.964 (95% CI 0.916-1.000).

### The SHAP to the Test Model Interpretation

**Figure 6.**
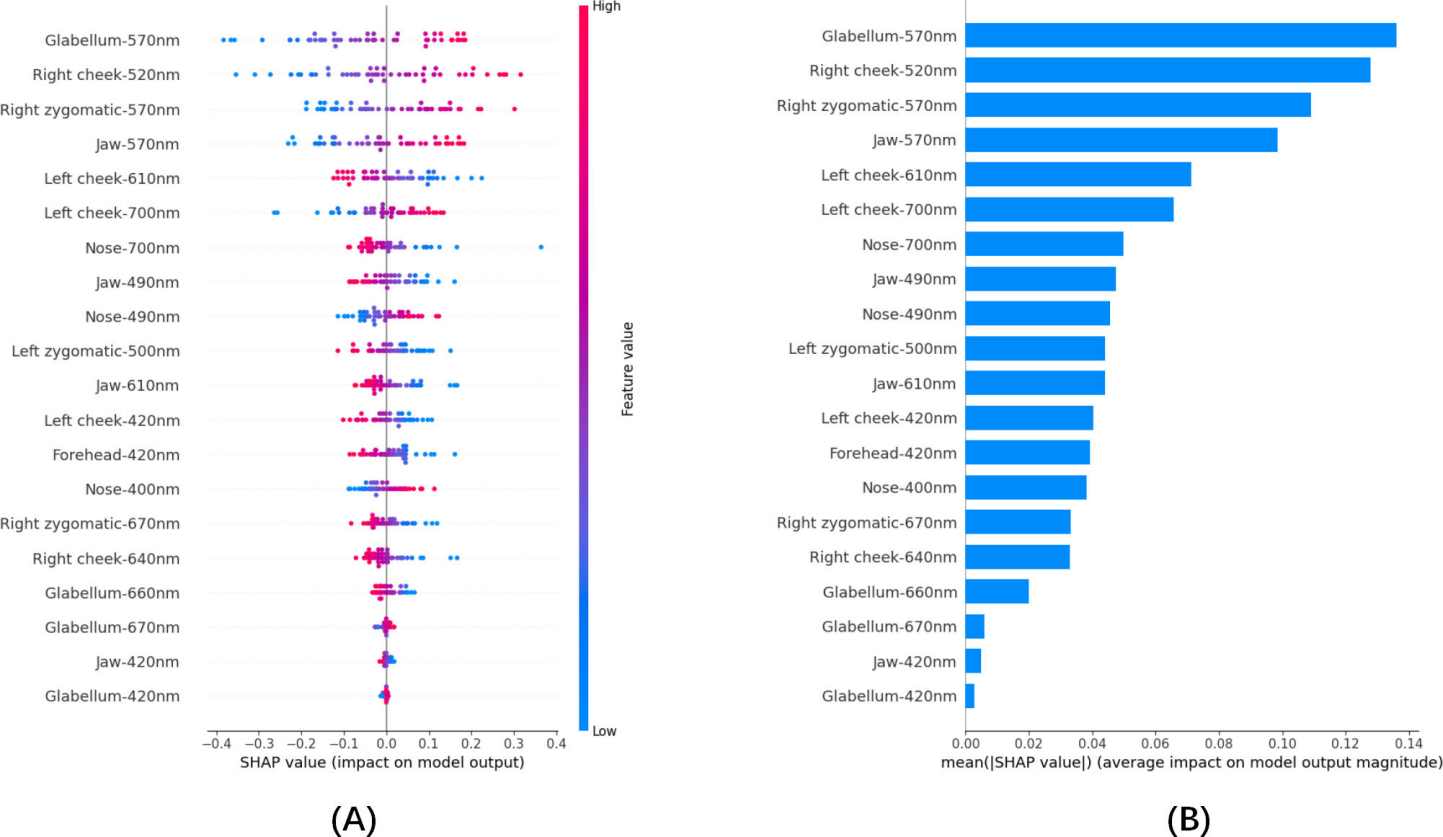
SHAP interprets the model. (**A**) Attributes of characteristics in SHAP. Each line represents a feature, and the abscissa is the SHAP value. Red dots represent higher eigenvalues and blue dots represent lower eigenvalues. (**B**) Feature importance ranking as indicated by SHAP. The matrix diagram describes the importance of each covariate in the development of the final prediction model. SHAP: Shapley additive explanations.

Since the SVM classification produced the best results, to intuitively interpret the selected variables, we used SHAP to illustrate how these variables contribute to the diagnosis of anemia within the SVM algorithm [49]. Figure 6A displays the 20 features extracted by LASSO regression. The vertical axis orders the features according to the sum of SHAP values for all samples, and the horizontal axis represents the SHAP values (the distribution of the feature’s impact on the model’s output); each point represents a sample, with the sample size stacked vertically. In each feature importance line, the corresponding results for all subjects are plotted with points of different colors, where red points denote high-risk values and blue points denote low-risk values. The results indicate that glabellum-570 nm, right cheek-520 nm, right zygomatic-570 nm, jaw-570 nm, left cheek-700 nm, nose-490 nm, nose-400 nm, glabellum-670 nm, and glabellum-420 nm positively impacted the diagnosis of anemia, while left cheek-610 nm, nose-700 nm, jaw-490 nm, left zygomatic-500 nm, jaw-610 nm, left cheek-420 nm, forehead-420 nm, right zygomatic-670 nm, right cheek-640 nm, glabellum-660 nm, and jaw-420 nm negatively impacted the diagnosis of anemia. Figure 6B shows the ranking of the 20 risk factors assessed by the average absolute SHAP values, with the x-axis representing SHAP values that indicate the importance of the predictive model.

## Discussion

### Principal Results

The findings of this study revealed that the spectral reflectance at most characteristic facial sites and bands was significantly higher in the anemia group than in the healthy control group, with particularly notable increases at some specific sites and bands. Incorporating facial spectral data into the modeling dataset facilitates the creation of an improved classification model, building upon previous studies.

As depicted in Figure 2, reflectance at forehead 410‐600 nm is higher in patients with anemia than in healthy controls. For the glabellum, nose, right zygomatic, right cheek, and left cheek, the spectral reflectance of patients with anemia from 400‐700 nm is higher than that of healthy controls. Reflectance at jaw 430‐610 nm, left zygomatic 400‐540 nm, and 510‐600 nm are higher than that of healthy controls. This study reveals a statistically significant increase in facial spectral reflectance among individuals with anemia compared to healthy controls. Several physiological mechanisms may contribute to the observed increase in reflectance.

First, changes in Hb concentration are a critical factor [50]. Hemoglobin, a light-absorbing component of blood, has a decrease in concentration with anemia, potentially enhancing light reflectance through tissues at specific wavelengths. This is particularly noticeable in the nose region. Second, alterations in blood oxygen saturation could play a role [51]. Reduced oxygen binding to Hb in anemic conditions could diminish the light absorption capacity, thereby increasing reflectance at certain wavelengths. It is feasible to use spectroscopy to detect skin oxygen saturation [52]. Additionally, anemia may induce changes in tissue moisture content, which in turn can modulate the optical behavior of tissues and influence light reflectance. The intensity of the absorption spectrum is proportional to the moisture content of the skin [53].

We used LASSO regression analysis to identify the 217 independent variables that showed statistically significant differences. This approach allowed us to compress variable coefficients and address collinearity issues, ultimately reducing the number of independent variables to 20. The selected variables, such as glabellum-570 nm, right cheek-520 nm, and left cheek-700 nm, highlight specific facial regions and wavelength bands that are associated with anemia risk. These findings suggest that certain spectral characteristics of the face may serve as indicators of anemia, warranting further investigation into the underlying physiological mechanisms.

To comprehensively analyze the classifiers in a multimodel comparison, we used 10 different ML algorithms, including logistic regression, decision tree, SVM, random forest, KNN, ANN, Bayesian classifier, XGBoost, adaptive boosting, and LightGBM. The performance of these models was evaluated using 2 distinct test sets, and the results indicated that the SVM prediction model demonstrated the best overall performance.

Within the SVM algorithm, we used the SHAP method to further explain the selected variables and their contribution to the diagnosis of anemia. The SHAP values provided intuitive insights into the impact of each feature on the model’s output. For instance, glabellum-420 nm, 570 nm, and 670 nm; nose-400 nm and 490 nm; jaw-570 nm; right cheek-520 nm; right zygomatic-570 nm; and left cheek-700 nm positively impacted the diagnosis of anemia, while glabellum-660nm; forehead-420nm; jaw-420, 490, and 610 nm; nose-700 nm; right zygomatic-670 nm; left zygomatic-500nm; right cheek-640 nm; and left cheek-420 and 610 nm negatively impacted the diagnosis. These findings suggested that specific spectral characteristics may play a crucial role in distinguishing between anemic and healthy individuals.

### Application and Advantages of Noninvasive Spectral Techniques in Anemia Diagnosis

According to the final model of this study, anemia was found to be strongly correlated with facial spectral reflectance at specific locations and bands. Therefore, incorporating facial spectral information into the modeling dataset can create a better classification model based on previous studies.

A plethora of studies have integrated advanced ML algorithms with spectral data, resulting in the development of predictive models that not only diagnose anemia but also predict the risk of disease onset in vulnerable populations. Noninvasive Hb measurement techniques have been developed to assess various sites on the human body, including fingers, palpebral conjunctiva, nail beds, bulbar conjunctiva, and other localized skin [32]. The ability to monitor Hb levels without the need for invasive procedures or complex equipment is expected to greatly enhance health care delivery in various settings, thereby contributing to the global effort to reduce the burden of anemia.

The face is not only the most exposed and conveniently assessable part of the human body but also holds significant importance in TCM diagnostics, where facial diagnosis plays a crucial role.

This study has unique advantages when targeting specific populations and environments. For particular groups, such as patients with anemia with low Hb levels, frequent invasive testing methods can lead to additional blood loss, therefore, using noninvasive spectroscopic detection technology can reduce the risk of blood loss. In specific environmental conditions, such as health check-ups for older individuals in the community, noninvasive spectral detection methods demonstrate their convenience and speed, with lower skill requirements for operators. Furthermore, in environments with a high incidence of infectious diseases, the implementation of noninvasive testing methods can significantly reduce the risk of group infections compared to traditional invasive testing techniques.

Based on the model of this study, strong correlations exist for facial spectral reflectance at specific locations and bands. Consequently, incorporating facial spectral information into the modeling dataset, building upon previous research, allows for the creation of an improved classification model. Given that pallor is often a common symptom of anemia [29,54-56], and the face is the most directly exposed part of the body, it serves as an important diagnostic tool. It is an important and intuitive diagnostic method. Therefore, it is necessary to establish a predictive model for anemia risk based on facial visible light reflection spectra. Future research will explore transitioning from contact spectroscopy to noncontact hyperspectral devices.

### Limitations

This study possesses several limitations. First, the sample size is small, necessitating future studies to use larger, multicenter cohorts. Second, the study’s focus on the middle-aged and older adult population suggests a need for future research to broaden the age range to enhance generalizability. Last, while facial coloration is influenced by various factors, this study controlled for common medical research variables. For the anemia group, additional physiological and biochemical markers that could affect facial coloration have not been considered. Future studies should aim to exclude the influence of such markers, including bilirubin, to provide a more comprehensive understanding of anemia-related facial spectral reflectance.

### Conclusion

Facial spectral data of patients with anemia showed distinctive features and correlations. These indicators were of greater significance for the differential diagnosis of anemia, including specific bands such as the glabellum at 570 nm, the nose at 400 nm, the right zygomatic at 400 nm, the right cheek at 530 nm, the left zygomatic at 400 nm, and the left cheek at 700 nm. They emphasized the subtle differences in light absorption and reflection associated with different degrees of anemia. In conclusion, this study constructed a predictive model based on the ML model, and the SVM model demonstrated superior performance in this study. In addition, we provided a personalized risk assessment for the development of patients with anemia explained by SHAP. This effective computer-aided approach can help first-line clinicians and patients identify and intervene early in the case of anemia. It holds promise for future applications in noninvasive diagnosis and population screening for anemia-related conditions.

## Supplementary material

10.2196/64204 Multimedia Appendix 1 Three-line table for all subjects.

## Abbreviations

AdaBoost: adaptive boosting
ANN: artificial neural network
AP: average precision
AUC: area under the receiver operating characteristic curve
DCA: decision curve analysis
DT: decision tree
Hb: hemoglobin
KNN: k-nearest neighbor
LASSO: least absolute shrinkage and selection operator
LightGBM: light gradient boosting machine
LR: logistic regression
ML: machine learning
NPV: negative predictive value
PPV: positive predictive value
PR: precision-recall
RF: random forest
ROC: receiver operating characteristic
SHAP: Shapley additive explanations
SVM: support vector machine
TCM: traditional Chinese medicine
XGBoost: Extreme Gradient Boosting
