# Supplementary material for: A Risk Warning Model for Anemia Based on Facial Visible Light Reflectance Spectroscopy: Cross-Sectional Study
Source: JMIR Med Inform. 2025 Feb 14;13:e64204. doi: 10.2196/64204 (PMC11845237; doi:10.2196/64204)
Supplement: Multimedia Appendix 1 [file medinform-v13-e64204-s001.docx]

| **Facial sites and bands** | **Anemia patients** | **Healthy controls** | ***t/Z*** | ***P*** |
| --- | --- | --- | --- | --- |
| Forehead400nm | 9.575(8.458-11.385) | 8.720(7.870-10.610) | -2.297 | .02 |
| Forehead640nm | 38.145(34.265-43.255) | 38.665(34.295-41.983) | -0.626 | .53 |
| Forehead650nm | 39.305(35.355-44.208) | 39.785(35.303-43.300) | -0.544 | .59 |
| Forehead660nm | 40.660(36.218-45.098) | 40.430(36.365-44.308) | -0.594 | .55 |
| Forehead670nm | 42.275(37.470-46.305) | 41.895(37.920-45.173) | -0.815 | .42 |
| Forehead680nm | 43.160(38.003-47.298) | 42.920(38.948-45.843) | -0.803 | .42 |
| Forehead690nm | 43.465(38.685-48.115) | 43.835(39.645-46.405) | -0.783 | .43 |
| Forehead700nm | 43.445(39.485-48.258) | 44.295(40.103-46.815) | -0.728 | .47 |
| Glabellum580nm | 27.135(23.768-30.485) | 22.360(20.000-24.655) | -6.121 | <.001 |
| Glabellum600nm | 32.850(29.273-37.550) | 29.435(26.765-33.128) | -4.381 | <.001 |
| Glabellum610nm | 36.920(32.860-41.350) | 33.875(31.120-37.810) | -3.435 | .001 |
| Glabellum620nm | 38.785(34.698-43.110) | 36.015(33.218-39.990) | -2.969 | .003 |
| Glabellum630nm | 40.170(35.807-44.570) | 37.495(34.360-41.518) | -2.713 | .007 |
| Glabellum640nm | 41.455(36.550-45.560) | 38.675(35.538-42.783) | -2.502 | .01 |
| Glabellum650nm | 42.460(37.368-46.493) | 39.765(36.528-44.075) | -2.446 | .01 |
| Glabellum660nm | 43.475(38.143-47.383) | 40.590(37.203-45.010) | -2.382 | .02 |
| Glabellum670nm | 44.655(39.255-48.605) | 41.900(37.225-46.285) | -2.407 | .02 |
| Glabellum680nm | 45.360(39.973-49.410) | 43.120(37.620-46.970) | -2.352 | .02 |
| Glabellum690nm | 45.815(40.535-49.998) | 44.200(38.273-47.565) | -2.343 | .02 |
| Glabellum700nm | 46.130(40.718-49.983) | 44.765(38.805-47.670) | -2.199 | .03 |
| Nose440nm | 15.145(12.700-16.938) | 11.965(10.67-13.845) | -5.797 | <.001 |
| Nose490nm | 20.460(17.330-22.368) | 16.490(14.643-18.590) | -5.691 | <.001 |
| Nose570nm | 23.745(20.915-26.453) | 19.435(17.203-21.825) | -6.265 | <.001 |
| Nose600nm | 34.350(29.500-36.593) | 29.800(26.870-31.840) | -4.959 | <.001 |
| Nose610nm | 39.015(32.115-41.895) | 34.700(31.393-37.340) | -4.128 | <.001 |
| Nose620nm | 40.955(34.130-44.240) | 37.220(33.700-39.870) | -3.626 | <.001 |
| Nose630nm | 42.545(36.110-46.030) | 38.700(35.413-41.690) | -3.415 | .001 |
| Nose640nm | 43.745(37.740-47.233) | 39.975(36.555-43.085) | -3.346 | .001 |
| Nose650nm | 44.715(38.843-48.450) | 41.190(37.778-44.358) | -3.156 | .002 |
| Nose660nm | 46.000(39.900-49.515) | 42.060(38.895-45.748) | -3.071 | .002 |
| Nose670nm | 47.420(40.750-51.055) | 43.385(40.550-47.333) | -3.054 | .002 |
| Nose680nm | 48.555(41.698-51.995) | 44.330(41.778-48.545) | -3.050 | .002 |
| Nose690nm | 49.585(42.225-52.800) | 45.160(42.368-49.198) | -2.992 | .003 |
| Nose700nm | 49.985(42.455-52.740) | 46.005(42.908-49.680) | -2.924 | .003 |
| Jaw400nm | 10.710(9.588-12.393) | 9.595(8.383-10.993) | -3.410 | .001 |
| Jaw410nm | 11.520(10.608-13.343) | 10.385(9.100-11.833) | -3.451 | .001 |
| Jaw420nm | 11.445(10.405-12.693) | 10.290(8.948-11.555) | -3.183 | .001 |
| Jaw430nm | 13.785(12.563-15.793) | 12.415(10.900-14.058) | -3.532 | <.001 |
| Jaw450nm | 16.200(15.378-19.023) | 15.050(13.193-16.953) | -3.585 | <.001 |
| Jaw470nm | 18.155(16.968-21.110) | 16.775(14.513-18.720) | -3.353 | .001 |
| Jaw480nm | 19.150(17.798-22.003) | 17.530(15.288-19.603) | -3.270 | .001 |
| Jaw490nm | 19.640(18.278-22.340) | 17.995(15.738-20.050) | -3.062 | .002 |
| Jaw500nm | 20.725(19.410-23.953) | 18.960(16.095-21.145) | -3.266 | .001 |
| Jaw600nm | 33.410(30.355-37.268) | 31.845(26.145-34.345) | -3.147 | .002 |
| Jaw610nm | 37.915(33.998-41.820) | 36.855(30.505-39.655) | -2.183 | .03 |
| Jaw620nm | 40.570(36.040-44.240) | 39.180(32.753-42.188) | -1.854 | .06 |
| Jaw630nm | 42.180(37.598-45.435) | 41.025(34.660-43.960) | -1.689 | .09 |
| Jaw640nm | 43.370(38.893-46.375) | 42.460(36.140-45.070) | -1.549 | .12 |
| Jaw650nm | 44.365(40.053-47.295) | 43.385(37.555-46.180) | -1.393 | .16 |
| Jaw660nm | 45.175(41.143-48.428) | 44.430(38.720-47.280) | -1.299 | .19 |
| Jaw670nm | 46.160(42.140-49.378) | 45.690(39.983-48.540) | -1.092 | .28 |
| Jaw680nm | 47.150(42.875-50.203) | 46.680(40.920-49.518) | -1.145 | .25 |
| Jaw690nm | 48.080(43.713-51.060) | 47.555(41.788-50.495) | -1.184 | .24 |
| Jaw700nm | 48.525(44.118-51.430) | 47.605(42.348-50.763) | -1.060 | .29 |
| Right zygomatic610nm | 37.905(34.463-40.960) | 34.495(29.638-37.398) | -3.941 | <.001 |
| Right zygomatic620nm | 39.915(35.118-43.028) | 36.795(31.595-39.975) | -3.546 | <.001 |
| Right zygomatic630nm | 41.200(37.373-44.105) | 38.235(33.368-41.585) | -3.289 | .001 |
| Right zygomatic640nm | 42.130(38.245-44.833) | 39.355(34.820-42.940) | -3.199 | .001 |
| Right zygomatic650nm | 43.340(39.633-45.798) | 40.620(36.220-44.143) | -3.126 | .002 |
| Right zygomatic660nm | 44.505(40.32-46.975) | 41.860(37.523-45.125) | -3.140 | .002 |
| Right zygomatic670nm | 45.975(41.668-47.948) | 43.475(39.058-46.250) | -3.108 | .002 |
| Right zygomatic680nm | 47.125(42.56-48.883) | 44.375(40.185-47.075) | -3.254 | .001 |
| Right zygomatic690nm | 47.805(43.625-49.583) | 45.080(41.038-47.783) | -3.300 | .001 |
| Right zygomatic700nm | 48.025(44.698-49.715) | 45.475(41.625-47.995) | -3.373 | .001 |
| Left zygomatic400nm | 10.895(9.118-12.713) | 9.625(8.443-11.365) | -2.364 | .02 |
| Left zygomatic410nm | 11.930(9.928-13.830) | 10.380(9.235-12.463) | -2.327 | .02 |
| Left zygomatic420nm | 11.880(10.028-13.800) | 10.450(9.338-12.543) | -2.235 | .03 |
| Left zygomatic430nm | 14.100(11.755-16.115) | 12.505(10.925-14.750) | -2.261 | .02 |
| Left zygomatic440nm | 15.365(12.770-17.435) | 13.805(11.913-16.260) | -2.189 | .03 |
| Left zygomatic450nm | 16.800(14.090-19.080) | 15.115(13.068-17.905) | -2.153 | .03 |
| Left zygomatic460nm | 17.690(14.928-20.073) | 16.000(13.905-18.918) | -2.132 | .03 |
| Left zygomatic470nm | 18.575(15.735-20.940) | 16.950(14.805-19.880) | -2.105 | .04 |
| Left zygomatic480nm | 19.460(16.650-21.700) | 17.925(15.605-20.798) | -2.136 | .03 |
| Left zygomatic490nm | 20.170(17.393-22.395) | 18.550(16.290-21.408) | -2.159 | .03 |
| Left zygomatic500nm | 21.455(18.423-23.908) | 19.545(17.093-22.610) | -2.327 | .02 |
| Left zygomatic540nm | 23.615(20.330-26.885) | 20.135(18.325-23.613) | -4.016 | <.001 |
| Left zygomatic680nm | 47.035(44.028-49.860) | 46.575(44.208-48.363) | -1.037 | .30 |
| Left zygomatic690nm | 47.790(44.880-50.265) | 47.185(45.103-48.905) | -1.108 | .27 |
| Left zygomatic700nm | 47.850(45.293-50.140) | 47.505(45.413-49.078) | -1.099 | .27 |
| Right cheeck590nm | 30.740(27.675-34.550) | 26.525(22.730-28.795) | -5.930 | <.001 |
| Right cheeck620nm | 39.835(37.008-43.473) | 37.145(33.785-39.745) | -3.686 | <.001 |
| Right cheeck640nm | 41.955(37.915-45.813) | 39.780(36.530-42.075) | -2.848 | .004 |
| Right cheeck650nm | 43.080(38.925-46.935) | 40.975(37.960-43.265) | -2.775 | .006 |
| Right cheeck660nm | 44.360(40.270-47.703) | 42.305(39.080-44.325) | -2.834 | .005 |
| Right cheeck670nm | 45.610(42.100-48.645) | 43.710(40.610-45.865) | -3.066 | .002 |
| Right cheeck680nm | 46.335(43.025-49.448) | 44.680(41.710-46.825) | -2.990 | .003 |
| Right cheeck690nm | 47.050(43.858-50.055) | 45.500(42.705-47.440) | -2.972 | .003 |
| Right cheeck700nm | 47.240(44.420-50.308) | 45.870(42.938-47.788) | -2.990 | .003 |
| Left cheeck400nm | 10.795(9.610-12.748) | 9.550(8.435-10.960) | -3.770 | <.001 |
| Left cheeck410nm | 11.665(10.535-13.890) | 10.335(9.183-12.028) | -3.716 | <.001 |
| Left cheeck430nm | 13.760(12.495-16.535) | 12.130(10.848-14.293) | -3.743 | <.001 |
| Left cheeck500nm | 21.695(19.403-24.550) | 19.150(17.288-22.053) | -3.732 | <.001 |
| Left cheeck540nm | 23.860(21.870-26.365) | 20.495(18.148-22.858) | -5.148 | <.001 |
| Left cheeck660nm | 45.465(42.705-47.655) | 44.040(41.290-45.925) | -2.327 | .02 |
| Left cheeck670nm | 46.650(44.313-48.86) | 45.440(42.480-47.275) | -2.485 | .01 |
| Left cheeck680nm | 47.495(45.393-49.55) | 46.365(43.220-48.138) | -2.591 | .01 |
| Left cheeck690nm | 48.050(46.193-50.19) | 47.170(44.155-48.580) | -2.600 | .009 |
| Left cheeck700nm | 48.175(46.383-50.295) | 47.490(44.665-48.813) | -2.556 | .01 |
| Forehead410nm | 10.615(2.212) | 9.794(1.722) | 2.585 | .01 |
| Forehead420nm | 10.583(2.144) | 9.763(1.664) | 2.669 | .008 |
| Forehead430nm | 12.531(2.589) | 11.537(2.064) | 2.649 | .009 |
| Forehead440nm | 13.670(2.819) | 12.564(2.281) | 2.696 | .008 |
| Forehead450nm | 14.987(3.085) | 13.746(2.527) | 2.747 | .007 |
| Forehead460nm | 15.773(3.185) | 14.499(2.672) | 2.705 | .008 |
| Forehead470nm | 16.539(3.254) | 15.257(2.813) | 2.632 | .009 |
| Forehead480nm | 17.333(3.333) | 16.028(2.942) | 2.593 | .01 |
| Forehead490nm | 17.938(3.412) | 16.565(2.990) | 2.673 | .008 |
| Forehead500nm | 19.043(3.559) | 17.535(3.127) | 2.812 | .006 |
| Forehead510nm | 20.030(3.708) | 18.309(3.224) | 3.094 | .002 |
| Forehead520nm | 20.638(3.831) | 18.619(3.204) | 3.571 | <.001 |
| Forehead530nm | 20.868(3.932) | 18.507(3.093) | 4.167 | <.001 |
| Forehead540nm | 20.923(4.053) | 18.218(2.982) | 4.747 | <.001 |
| Forehead550nm | 21.432(3.831) | 18.638(3.124) | 4.992 | <.001 |
| Forehead560nm | 22.212(3.925) | 19.267(3.187) | 5.144 | <.001 |
| Forehead570nm | 23.724(4.132) | 20.675(3.325) | 5.077 | <.001 |
| Forehead580nm | 25.482(4.322) | 22.481(3.509) | 4.761 | <.001 |
| Forehead590nm | 27.927(4.561) | 25.219(3.801) | 4.029 | <.001 |
| Forehead600nm | 31.117(4.936) | 29.155(4.341) | 2.636 | .009 |
| Forehead610nm | 34.442(5.283) | 33.226(4.810) | 1.503 | .14 |
| Forehead620nm | 36.188(5.493) | 35.231(5.065) | 1.132 | .26 |
| Forehead630nm | 37.363(5.621) | 36.640(5.195) | 0.834 | .41 |
| Glabellum400nm | 10.954(2.201) | 9.276(1.857) | 5.146 | <.001 |
| Glabellum410nm | 11.763(2.318) | 10.003(1.985) | 5.094 | <.001 |
| Glabellum420nm | 11.580(2.261) | 9.916(1.964) | 4.906 | <.001 |
| Glabellum430nm | 13.886(2.704) | 11.802(2.352) | 5.136 | <.001 |
| Glabellum440nm | 15.130(2.934) | 12.864(2.587) | 5.116 | <.001 |
| Glabellum450nm | 16.555(3.192) | 14.074(2.858) | 5.113 | <.001 |
| Glabellum460nm | 17.399(3.336) | 14.814(2.998) | 5.090 | <.001 |
| Glabellum470nm | 18.212(3.460) | 15.547(3.123) | 5.048 | <.001 |
| Glabellum480nm | 19.049(3.559) | 16.299(3.242) | 5.045 | <.001 |
| Glabellum490nm | 19.646(3.598) | 16.839(3.294) | 5.082 | <.001 |
| Glabellum500nm | 20.827(3.686) | 17.768(3.478) | 5.331 | <.001 |
| Glabellum510nm | 21.846(3.777) | 18.394(3.670) | 5.789 | <.001 |
| Glabellum520nm | 22.346(3.876) | 18.545(3.758) | 6.220 | <.001 |
| Glabellum530nm | 22.342(4.000) | 18.278(3.762) | 6.536 | <.001 |
| Glabellum540nm | 22.133(4.098) | 17.836(3.746) | 6.837 | <.001 |
| Glabellum550nm | 22.565(4.086) | 18.243(3.777) | 6.860 | <.001 |
| Glabellum560nm | 23.348(4.097) | 18.755(3.911) | 7.163 | <.001 |
| Glabellum570nm | 24.982(4.231) | 20.065(4.160) | 7.319 | <.001 |
| Glabellum580nm | 26.873(4.508) | 21.886(4.412) | 6.982 | <.001 |
| Glabellum590nm | 29.521(4.935) | 24.751(4.772) | 6.136 | <.001 |
| Nose400nm | 10.515(2.394) | 8.457(1.439) | 6.505 | <.001 |
| Nose410nm | 11.292(2.500) | 9.148(1.534) | 6.457 | <.001 |
| Nose420nm | 10.981(2.515) | 8.994(1.511) | 5.983 | <.001 |
| Nose430nm | 13.506(2.920) | 10.954(1.856) | 6.515 | <.001 |
| Nose450nm | 16.359(3.489) | 13.320(2.354) | 6.377 | <.001 |
| Nose460nm | 17.293(3.639) | 14.126(2.517) | 6.322 | <.001 |
| Nose470nm | 18.214(3.779) | 14.949(2.669) | 6.233 | <.001 |
| Nose480nm | 19.137(3.909) | 15.780(2.783) | 6.177 | <.001 |
| Nose500nm | 20.894(4.090) | 17.313(2.870) | 6.330 | <.001 |
| Nose510nm | 21.786(4.168) | 17.954(3.005) | 6.587 | <.001 |
| Nose520nm | 22.109(4.180) | 18.076(3.085) | 6.856 | <.001 |
| Nose530nm | 21.918(4.102) | 17.749(3.135) | 7.133 | <.001 |
| Nose540nm | 21.513(4.100) | 17.244(3.184) | 7.263 | <.001 |
| Nose550nm | 21.77(4.304) | 17.546(3.186) | 6.967 | <.001 |
| Nose560nm | 22.419(4.362) | 18.074(3.216) | 7.079 | <.001 |
| Nose580nm | 26.048(4.603) | 21.394(3.503) | 7.107 | <.001 |
| Nose590nm | 29.09(4.808) | 24.514(3.758) | 6.623 | <.001 |
| Jaw440nm | 15.291(2.972) | 13.855(3.252) | 2.878 | .005 |
| Jaw460nm | 17.603(3.482) | 15.977(3.495) | 2.911 | .004 |
| Jaw510nm | 21.891(4.222) | 19.645(3.956) | 3.429 | .001 |
| Jaw520nm | 22.354(4.320) | 19.738(4.017) | 3.917 | <.001 |
| Jaw530nm | 22.228(4.521) | 19.394(4.014) | 4.140 | <.001 |
| Jaw540nm | 21.786(4.838) | 18.836(4.000) | 4.151 | <.001 |
| Jaw550nm | 22.419(4.408) | 19.057(3.917) | 5.035 | <.001 |
| Jaw560nm | 23.042(4.498) | 19.465(4.025) | 5.234 | <.001 |
| Jaw570nm | 24.492(4.721) | 20.713(4.274) | 5.241 | <.001 |
| Jaw580nm | 26.351(4.892) | 22.534(4.514) | 5.064 | <.001 |
| Jaw590nm | 29.173(5.147) | 25.600(4.779) | 4.492 | <.001 |
| Right zygomatic400nm | 10.340(2.459) | 8.735(1.793) | 4.656 | <.001 |
| Right zygomatic410nm | 11.214(2.628) | 9.486(1.940) | 4.674 | <.001 |
| Right zygomatic420nm | 11.241(2.670) | 9.547(1.943) | 4.532 | <.001 |
| Right zygomatic430nm | 13.221(3.016) | 11.159(2.303) | 4.800 | <.001 |
| Right zygomatic440nm | 14.415(3.244) | 12.152(2.534) | 4.856 | <.001 |
| Right zygomatic450nm | 15.800(3.510) | 13.298(2.795) | 4.924 | <.001 |
| Right zygomatic460nm | 16.673(3.664) | 14.057(2.945) | 4.916 | <.001 |
| Right zygomatic470nm | 17.544(3.810) | 14.838(3.089) | 4.872 | <.001 |
| Right zygomatic480nm | 18.430(3.956) | 15.636(3.227) | 4.835 | <.001 |
| Right zygomatic490nm | 19.046(4.059) | 16.212(3.306) | 4.782 | <.001 |
| Right zygomatic500nm | 20.244(4.222) | 17.126(3.437) | 5.058 | <.001 |
| Right zygomatic510nm | 21.328(4.366) | 17.780(3.517) | 5.589 | <.001 |
| Right zygomatic520nm | 21.910(4.459) | 18.016(3.512) | 6.058 | <.001 |
| Right zygomatic530nm | 22.015(4.493) | 17.891(3.449) | 6.430 | <.001 |
| Right zygomatic540nm | 21.888(4.550) | 17.608(3.388) | 6.664 | <.001 |
| Right zygomatic550nm | 22.671(4.309) | 18.145(3.365) | 7.311 | <.001 |
| Right zygomatic560nm | 23.443(4.430) | 18.715(3.420) | 7.462 | <.001 |
| Right zygomatic570nm | 25.021(4.708) | 20.039(3.598) | 7.426 | <.001 |
| Right zygomatic580nm | 26.993(4.912) | 21.879(3.827) | 7.254 | <.001 |
| Right zygomatic590nm | 29.779(5.115) | 24.786(4.137) | 6.703 | <.001 |
| Right zygomatic600nm | 33.358(5.447) | 29.093(4.736) | 5.218 | <.001 |
| Left zygomatic510nm | 22.737(4.394) | 20.998(4.443) | 2.458 | .02 |
| Left zygomatic520nm | 23.443(4.509) | 21.356(4.449) | 2.909 | .004 |
| Left zygomatic530nm | 23.694(4.578) | 21.217(4.491) | 3.412 | .001 |
| Left zygomatic550nm | 24.372(4.637) | 21.392(4.739) | 3.969 | <.001 |
| Left zygomatic560nm | 25.208(4.728) | 22.050(4.718) | 4.176 | <.001 |
| Left zygomatic570nm | 26.852(4.894) | 23.563(4.732) | 4.267 | <.001 |
| Left zygomatic580nm | 28.795(4.991) | 25.537(4.826) | 4.144 | <.001 |
| Left zygomatic590nm | 31.483(5.044) | 28.521(4.920) | 3.712 | <.001 |
| Left zygomatic600nm | 34.949(5.124) | 32.766(5.074) | 2.674 | .008 |
| Left zygomatic610nm | 38.472(5.135) | 37.075(4.994) | 1.722 | .09 |
| Left zygomatic620nm | 40.311(5.073) | 39.23(4.922) | 1.350 | .18 |
| Left zygomatic630nm | 41.522(5.086) | 40.701(4.775) | 1.039 | .30 |
| Left zygomatic640nm | 42.391(5.174) | 41.78(4.674) | 0.773 | .44 |
| Left zygomatic650nm | 43.439(5.133) | 42.926(4.450) | 0.667 | .51 |
| Left zygomatic660nm | 44.454(5.034) | 43.968(4.162) | 0.657 | .51 |
| Left zygomatic670nm | 45.818(4.837) | 45.287(3.829) | 0.761 | .45 |
| Right cheeck400nm | 10.671(2.427) | 8.969(1.617) | 5.153 | <.001 |
| Right cheeck410nm | 11.592(2.598) | 9.759(1.773) | 5.147 | <.001 |
| Right cheeck420nm | 11.613(2.597) | 9.829(1.824) | 4.965 | <.001 |
| Right cheeck430nm | 13.706(3.025) | 11.505(2.095) | 5.281 | <.001 |
| Right cheeck440nm | 14.975(3.282) | 12.551(2.313) | 5.333 | <.001 |
| Right cheeck450nm | 16.443(3.571) | 13.761(2.567) | 5.387 | <.001 |
| Right cheeck460nm | 17.355(3.730) | 14.536(2.710) | 5.400 | <.001 |
| Right cheeck470nm | 18.245(3.882) | 15.327(2.839) | 5.358 | <.001 |
| Right cheeck480nm | 19.135(4.051) | 16.143(2.952) | 5.271 | <.001 |
| Right cheeck490nm | 19.703(4.283) | 16.753(2.993) | 4.986 | <.001 |
| Right cheeck500nm | 20.964(4.336) | 17.738(3.167) | 5.308 | <.001 |
| Right cheeck510nm | 22.203(4.295) | 18.461(3.353) | 6.065 | <.001 |
| Right cheeck520nm | 22.881(4.381) | 18.774(3.418) | 6.527 | <.001 |
| Right cheeck530nm | 23.022(4.547) | 18.72(3.359) | 6.722 | <.001 |
| Right cheeck540nm | 22.897(4.823) | 18.515(3.285) | 6.632 | <.001 |
| Right cheeck550nm | 23.692(4.668) | 19.177(3.419) | 6.892 | <.001 |
| Right cheeck560nm | 24.518(4.743) | 19.821(3.517) | 7.025 | <.001 |
| Right cheeck570nm | 26.149(4.887) | 21.202(3.729) | 7.107 | <.001 |
| Right cheeck580nm | 28.082(5.032) | 23.041(3.958) | 6.953 | <.001 |
| Right cheeck600nm | 34.155(5.420) | 30.056(4.480) | 5.148 | <.001 |
| Right cheeck610nm | 37.580(5.529) | 34.389(4.682) | 3.889 | <.001 |
| Right cheeck630nm | 40.511(5.616) | 37.994(4.926) | 2.976 | .003 |
| Left cheeck420nm | 12.165(2.784) | 10.779(2.058) | 3.535 | .001 |
| Left cheeck440nm | 15.701(3.555) | 13.860(2.660) | 3.664 | <.001 |
| Left cheeck450nm | 17.232(3.862) | 15.239(2.916) | 3.638 | <.001 |
| Left cheeck460nm | 18.157(3.995) | 16.104(3.027) | 3.616 | <.001 |
| Left cheeck470nm | 19.062(4.082) | 16.984(3.108) | 3.578 | <.001 |
| Left cheeck480nm | 19.997(4.146) | 17.902(3.185) | 3.539 | .001 |
| Left cheeck490nm | 20.691(4.144) | 18.603(3.226) | 3.512 | .001 |
| Left cheeck510nm | 23.137(4.391) | 20.612(3.475) | 3.982 | <.001 |
| Left cheeck520nm | 23.820(4.484) | 20.989(3.486) | 4.402 | <.001 |
| Left cheeck530nm | 24.033(4.558) | 20.912(3.416) | 4.839 | <.001 |
| Left cheeck550nm | 24.652(4.843) | 21.305(3.329) | 5.031 | <.001 |
| Left cheeck560nm | 25.536(4.884) | 22.039(3.378) | 5.202 | <.001 |
| Left cheeck570nm | 27.265(4.943) | 23.595(3.512) | 5.345 | <.001 |
| Left cheeck580nm | 29.261(4.986) | 25.569(3.633) | 5.287 | <.001 |
| Left cheeck590nm | 31.988(4.981) | 28.503(3.780) | 4.922 | <.001 |
| Left cheeck600nm | 35.474(4.991) | 32.729(4.063) | 3.766 | <.001 |
| Left cheeck610nm | 38.983(4.946) | 36.957(4.192) | 2.759 | .006 |
| Left cheeck620nm | 40.811(4.834) | 39.011(4.183) | 2.487 | .01 |
| Left cheeck630nm | 42.016(4.828) | 40.369(4.213) | 2.271 | .03 |
| Left cheeck640nm | 42.880(4.946) | 41.345(4.333) | 2.062 | .04 |
| Left cheeck650nm | 43.908(4.882) | 42.436(4.303) | 1.999 | .05 |
